# Experimental Study on the Uplift Bearing Mechanism of New Pneumatic Pipe Piles

**DOI:** 10.3390/ma18071414

**Published:** 2025-03-23

**Authors:** Huan Wang, Rui Zhang, Zhengnan Liu, Xiang Wang, Xiwei Zhang

**Affiliations:** 1 College of Traffic and Transportation Engineering, Changsha University of Science & Technology, Changsha 410114, China; whhh@stu.csust.edu.cn (H.W.); lzn@csust.edu.cn (Z.L.); zhangxiwei@stu.csust.edu.cn (X.Z.); 2 National Key Laboratory of Green and Long-Life Road Engineering in Extreme Environment (Changsha), Changsha University of Science & Technology, Changsha 410114, China; 3 Engineering Research Center of Catastrophic Prophylaxis and Treatment of Road & Traffic Safety of Ministry of Education, Changsha University of Science & Technology, Changsha 410114, China; 4 Hunan Communications Research Institute Co., Ltd., Changsha 410015, China; 5 China Railway Siyuan Survey and Design Group Co., Ltd., Wuhan 430063, China; wangxiangzt@126.com

**Keywords:** micropiles, uplift resistance, discrete element method, soil slope, field tests

## Abstract

To enhance the uplift resistance of micropiles used in soil slope reinforcement and ensure the stability and safety of slope structures, a novel micropile incorporating a small-scale pneumatic device and anchorage components was developed, and its uplift performance was evaluated. Through field uplift tests, the uplift load–vertical displacement relationship of the new micropile and conventional micropile in silty clay strata was compared. Numerical simulations were also conducted to reveal the uplift mechanism and analyze the influence of an anchorage component layout on the micropile’s uplift resistance. The field tests showed that the ultimate uplift capacity of a 3 m long novel micropile increased by 161.7% compared to that of a conventional micropile, with a 14.7% reduction in displacement. When the anchorage components were deployed without grouting, the novel micropile achieved 70.7% of the uplift capacity of a conventional micropile, indicating a certain level of uplift resistance. Numerical simulation results indicated that the novel micropile altered the stress state of the surrounding soil, and the anchorage components changed the load transfer mechanism during micropile uplift from vertical interfacial friction to a combination of anchorage pressure and soil friction, significantly enhancing uplift resistance. For an 8 m long micropile without anchorage components, the ultimate uplift capacity was 489.9 kN. With the addition of 1 m of anchorage length, the capacity increased to 661.5 kN, a 35.0% improvement. Subsequently, each additional meter of anchorage length increased the micropile’s capacity by 10.9% to 16.0%, with a cost increase of only 5.7%. The research findings provide valuable scientific references for the design and remediation of soil slope reinforcement.

## 1. Introduction

The construction of railways and highways in mountainous regions of China inevitably results in a large number of artificially excavated soil slopes. Under the influence of heavy or continuous rainfall, these slopes are prone to instability and require effective reinforcement. Soil nails and anchor rods are the most commonly used reinforcement elements in railway and highway slope stabilization [1,2,3,4]. However, when these elements are used to reinforce fine-grained soil slopes, the weak bonding and frictional resistance between the rod, grout, and soil can lead to reduced reinforcement effectiveness, resulting in structural failure and slope instability [5,6]. Research on new reinforcement elements to enhance their performance and reveal their mechanisms is of significant theoretical and engineering importance for ensuring slope safety and stability.

Numerous studies have been conducted by domestic and international scholars on existing slope reinforcement techniques. It is widely recognized that poor soil properties [7], insufficient pile side friction [8,9], inadequate grout material performance [10], construction quality issues [11], and environmental changes [12] are factors contributing to insufficient uplift resistance of reinforcement elements. In addition to these factors, the interaction between the rod, grout material, and soil also significantly affects the uplift capacity of the pile. Current techniques to enhance uplift resistance include expanded pile technology [13] and high-pressure jet grouting technology [14]. Expanded pile technology increases the uplift capacity by setting an enlarged head structure at the bottom or specific locations of the pile [15]. However, this technique is costly and can cause soil squeezing effects during the static pressure process, leading to issues such as floating piles. Jet grouting piles use high-speed rotating nozzles to inject grout material into the soil layer in the form of high-pressure jets, increasing the mixing degree and consolidation strength of the grout material and soil layer to enhance uplift capacity [16,17]. However, this technique has drawbacks, such as high construction difficulty, a significant impact on existing structures, severe environmental pollution, and unstable curing times. Although these common engineering methods can improve the uplift capacity of piles to some extent, they primarily enhance their strength by increasing the contact area and friction between the grout material and soil. This approach only strengthens a single interface and does not integrate the pile, grout material, and soil as a whole for combined reinforcement. In recent years, micropiles have been widely used in slope reinforcement due to their quick and flexible construction. Sabri M [18] studied micropile reinforcement of soil. Sun Shuwei [19] proposed an analytical theory for the ultimate resistance of micropile-reinforced soil slopes and optimized the micropile design method. Luo Hui [20] conducted in situ static load tests on micropiles and analyzed the load transfer and interaction mechanism between the pile and soil at the slope toe from the perspectives of pile length, load transfer, uplift deformation, pile axial force, and pile side friction. Ma Pengjie [21] verified the feasibility of micropile reinforcement for fine-grained soil slopes through model tests. These studies indicate that micropiles have good application prospects in slope reinforcement. However, due to their small diameter, the cross-sectional area of the pile is limited, resulting in relatively low uplift resistance when subjected to uplift loads. To enhance the overall reinforcement effect, it is usually necessary to increase the number of micropiles or their length.

Given the shortcomings of existing treatment methods, a new micropile treatment technology has been proposed. This technology integrates a small pneumatic device within the micropile cavity and connects it to a thin blade-shaped anchorage component. The retractable thin blade-shaped anchorage component integrates the micropile, grout material, and soil as a whole for combined reinforcement, thereby enhancing the uplift capacity of the micropile and achieving “pile–grout–soil integrated reinforcement.” Through field tests, the relationship between the load and displacement of the new micropile was revealed, and numerical simulations were used to analyze the influence of the anchorage component reinforcement length on the uplift performance of the micropile. The research findings provide references for the design and treatment of soil slope reinforcement.

## 2. Experimental Section

### 2.1. Structure of the New Micropile

**Figure 1:**
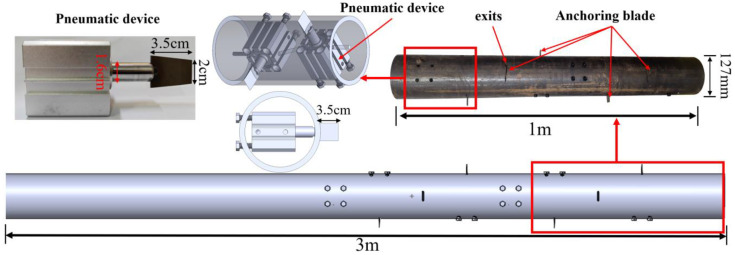
Composition of new micropile structure.

The structure of the new micropile is shown in Figure 1. It mainly consists of the micropile body, anchorage components, and a small pneumatic device. The front blade angle of the anchorage component is designed at 25°, and the pile wall is equipped with ejection ports every 10 cm to facilitate the extension of the anchorage components driven by the small pneumatic device. The front end of the pneumatic device is welded to the anchorage component, and the rear end is fixed to the micropile wall with positioning nuts. The air inlets of all pneumatic devices are connected to the same pipeline system, and the anchorage components are ejected by an air compressor. In the initial state, the tail end of the anchorage component is in contact with the small pneumatic device, and the front part of the anchorage component does not extend beyond the outer wall of the pile. In the working state, the small pneumatic device extends radially, pushing the anchorage component to extend radially from the ejection port and penetrate the soil, ultimately forming a stable anchorage section to exert uplift resistance.

### 2.2. Field Test Design of the New Micropile

To verify the uplift performance improvement of the new micropile, field uplift tests were conducted. The tests compared three conditions: the new micropile with ejected anchorage components and grouting, the new micropile with ejected anchorage components but no grouting, and the conventional micropile.

#### 2.2.1. Test Site

The test site was located on the campus of Changsha University of Science and Technology. Drilling data indicated that the site consists of brown-yellow soil from 0 to 10.0 m and highly weathered purple-red rock from 10.0 to 13.3 m. Since the new micropile is designed for soil slope reinforcement, the test was conducted based on soil characteristics. To verify the applicability of the novel micropile at this test site, an improved loading frame was used to conduct an indoor penetration test.

**Figure 2:**
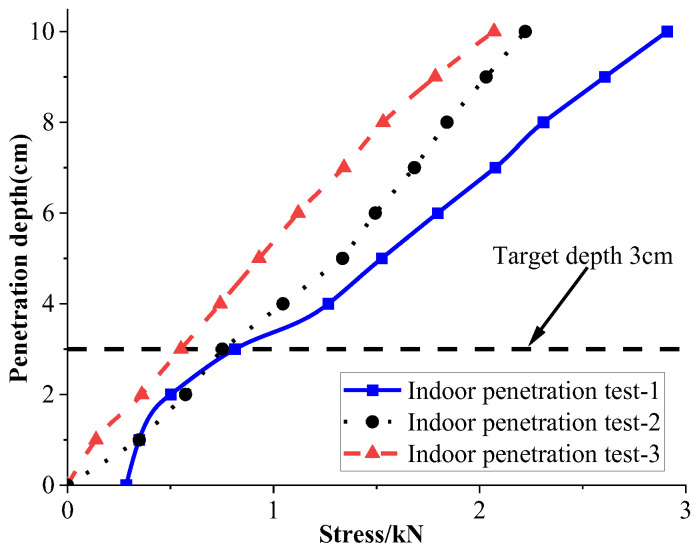
Relationship between load and penetration depth.

According to the test data provided by the manufacturer, the small pneumatic device has a pressure resistance limit of 1.5 MPa. When the pneumatic device operates at a pressure of 0.7 MPa, it can generate a thrust of 0.8 kN. After compacting the soil sample to its natural density, the relationship between the load and penetration depth was measured, as shown in Figure 2.

#### 2.2.2. Test Procedure

**Figure 3:**
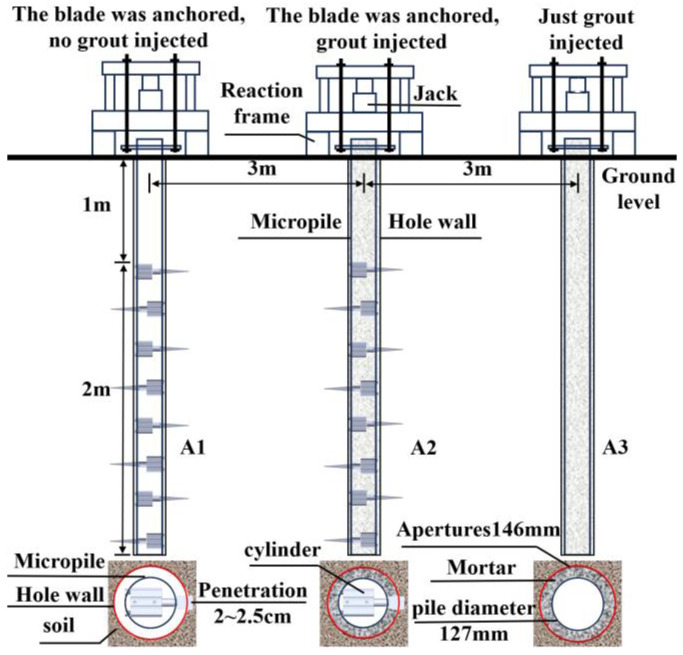
Layout of field test piles.

The test piles used in the field uplift tests were steel-pipe micropiles made of Q345 steel, with an outer diameter of 127.0 mm, a wall thickness of 4.5 mm, and a length of 3.0 m. The anchorage length of the anchorage components ranged from 1.0 to 3.0 m, with a spacing of 10.0 cm, and the borehole diameter was 146.0 mm. The tests compared the uplift resistance under three conditions: the new micropile with ejected anchorage components but no grouting (labeled A1), the new micropile with ejected anchorage components and grouting (labeled A2), and the conventional micropile with grouting (labeled A3). By comparing these conditions, the reinforcement effect of the new micropile before and after grouting was evaluated. The specific layout is shown in Figure 3.

The field test procedure is illustrated in Appendix A, with the specific steps as follows:

(1) **Drilling**: A geological drilling rig with water drilling was used to create boreholes with a diameter of 146.0 mm. Mud was used to stabilize the borehole walls and prevent collapse.
(2) **Micropile assembly and installation**: The small pneumatic device and anchorage components were installed inside the micropile. The micropile segments were connected via threaded joints. After drilling, the micropile was vertically lowered into the center of the borehole using the geological drilling rig. A centering bracket was welded at the top to ensure the steel-pipe micropile was securely and centrally installed.
(3) **Grouting**: The pneumatic devices were regularly arranged inside the micropile, leaving sufficient space for grouting before the cylinders were activated. Grouting was performed intermittently from the bottom up using a grouting pipe. The grouting material was PC42.5R cement with a water–cement ratio of 0.5, and the grouting pressure was set at 1.0 MPa.
(4) **Ejection of anchorage components**: The new micropile was placed into the borehole according to the predetermined anchorage positions. The air inlets of the small pneumatic devices were connected to the air compressor, and high-pressure gas was injected through the high-pressure inflation device to push the anchorage components into the soil.
(5) **Curing**: After grouting and ejecting the anchorage components, the micropile was left to cure for 28 days. Sampling tests showed that the compressive strength after 28 days of curing ranged from 32.4 to 38.6 MPa.
(6) **Loading**: The center of the jack base was aligned with the centroid of the test pile’s cross-section. The field test setup is shown in Appendix A. The load was applied incrementally at 0.1 times the predicted ultimate uplift capacity, with each load level maintained for 120 min until the test pile failed or further loading was impossible. The vertical displacement of the test pile was measured using two dial gauges with a range of 50 mm, symmetrically placed at the pile head, with a measurement accuracy of 0.01 mm.

#### 2.2.3. Numerical Simulation and Calibration Methods

Currently, the discrete element theory is widely applied in the study of the mechanical behavior of cohesive soils [22,23]. To explore the mechanism of the new micropile in improving uplift stability, this paper uses discrete element theory for numerical model calculation and analysis.

### 2.3. Selection of Microscopic Parameters and Model Construction

**Figure 4:**
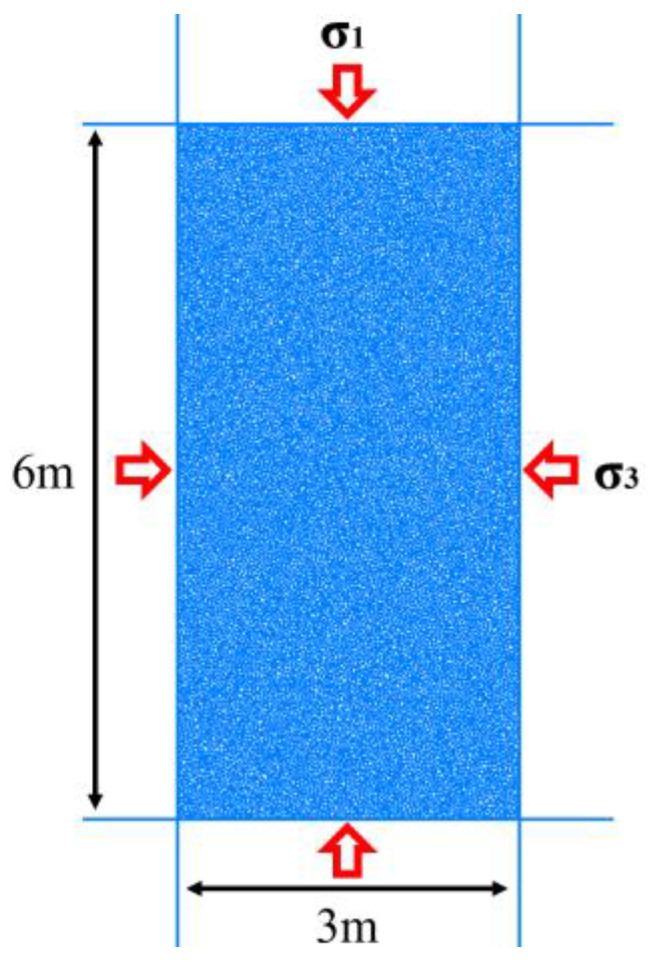
Biaxial compression test model.

A biaxial compression test was used to calibrate the microscopic parameters of the numerical model to ensure that the macroscopic properties were consistent with the actual properties of the field test site. The soil biaxial compression test model constructed in this paper is shown in Figure 4. The model dimensions are 3 m × 6 m (width × height), with particle sizes ranging from 0.016 to 0.024. The biaxial calibration model contains 15,019 particles.

In the model, the micropile interface was simulated using the parallel bond contact model. This interface model can resist and transfer forces and moments when bonded, exhibiting linear elastic behavior. When the interface force exceeds the strength limit, the bonding model fails, and the interface transitions to a linear contact model, which is used to simulate linear forces and damping forces. This modeling approach is more suitable for simulating the mechanical behavior of geotechnical materials [24].

When the interface force is relatively low and the bonding model remains intact, the interface interaction force, FcFcis, is calculated using Equation (1):

(1) $$\begin{array}{l} F_{C}=F^{1}+F^{d}+\bar{F}\\ M_{C}=\bar{M} \end{array}$$

In the equation,

*F^1^* represents the linear contact force, *F^d^* denotes the damping force, and *M_c_,* $\bar{M}$ refers to the bonding load.

When the interface force exceeds the strength limit, the bonded model fails, and if the bonding load *M_c_* decreases, the model transitions into a linear contact model. At this stage, the bonding load *M_c_* is reduced to zero.

## 3. Results and Discussion

### 3.1. Indoor Test Results and Discussion

Physical test results showed that the soil sample contained 72.12% coarse particles, 20.12% silt, and 7.76% clay. The detailed physical parameters are listed in Table 1, and the soil was classified as silty clay.

The results from three parallel indoor penetration tests (labeled as Tests 1, 2, and 3) indicate that under a thrust of 0.8 kN (0.7 MPa pressure), the pneumatic device can penetrate the soil to a depth of 3.0–4.5 cm, exceeding the predetermined penetration depth. This demonstrates that the pneumatic device possesses sufficient penetration capability even under relatively low-pressure conditions. Since the piston-type air compressor used on-site can reach a pressure of 1.25 MPa, it is capable of generating greater thrust, fully meeting the penetration requirements for the tests.

### 3.2. Field Test Results and Discussion

**Figure 5:**
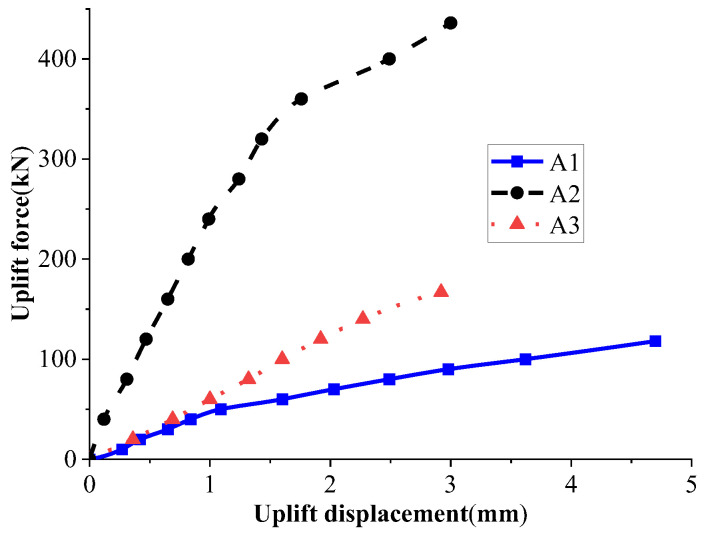
Curve of vertical load versus uplift displacement.

The uplift performance of the new micropile under three different conditions was compared: ejected anchorage components without grouting (A1), ejected anchorage components with grouting (A2), and the conventional micropile with grouting (A3). Through the tests, the load–displacement relationship curves of the new micropile and conventional micropile before and after grouting were obtained, as shown in Figure 5.

From Figure 5, it can be seen that as the uplift load increased, the uplift displacement increased nonlinearly, with a slow initial growth rate followed by a slightly accelerated trend. This trend was more pronounced in the new micropile. According to the “Technical Code for Testing of Building Foundation Piles” [25], when the uplift curve exhibits a gradual change characteristic, the maximum load value is taken as the ultimate uplift capacity. Therefore, when only the anchorage components were ejected without grouting (A1), the ultimate uplift capacity of the micropile was 118 kN. When the anchorage components were ejected and grouting was completed (A2), the ultimate uplift capacity of the micropile was 436 kN. For the conventional micropile with grouting (A3), the ultimate uplift capacity was 167 kN. The comparison shows that the uplift performance of the new micropile was significantly higher than that of the conventional micropile. In the ultimate state, the ultimate uplift capacity of the new micropile increased by 161.7% compared to that of the conventional micropile, and the displacement was reduced by 14.7%. Additionally, when only the anchorage components were ejected without grouting, the ultimate uplift capacity of the new micropile reached 70.7% of that of the conventional micropile, indicating a certain level of uplift resistance. By observing the characteristics of the uplift curves, the uplift process can be divided into three stages: the elastic linear deformation stage, the elastic–plastic deformation stage, and the failure stage. The new micropile significantly increased the uplift resistance in the elastic linear deformation stage. This is because the new micropile, under the action of the “—”-shaped anchorage components, integrates the micropile, grout material, and soil into a whole. When the soil and grout above the anchorage components have not yet undergone compressive failure, the pile body remains in the elastic deformation stage for a long time. As the uplift load continues to increase, the soil and grout above the anchorage components undergo compressive deformation, gradually transitioning to the elastic–plastic deformation stage, ultimately leading to the failure of the micropile.

In summary, the uplift resistance and deformation control ability of the new micropile have been significantly improved. This improvement is mainly due to the close integration of the “—”-shaped anchorage components, micropile, grout material, and soil. This integration improves upon the traditional conventional micropile, which relies solely on interfacial friction to provide anchorage force. Instead, the new micropile provides higher uplift resistance through the compressive resistance of the soil and grout above the “—”-shaped anchorage components and the interfacial friction of the micropile itself.

### 3.3. Micro-Parameter Calibration Results

**Figure 6:**
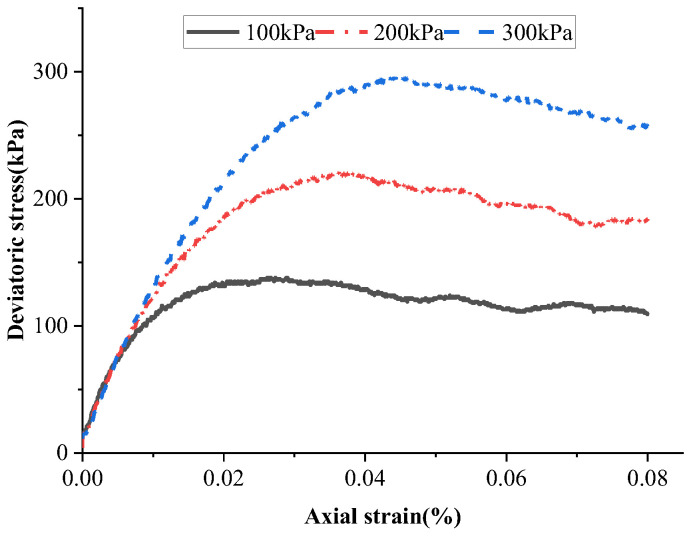
Deviator stress–axial strain curve.

The parallel bond model was used for particle contact, as it can accurately reflect the shear characteristics of clay materials. After extensive trial calculations using the trial-and-error method, the microscopic parameters listed in Table 2 were selected for the simulation. Biaxial compression tests were conducted under confining pressures of 100, 200, and 300 kPa, with corresponding maximum deviator stresses of 137, 220, and 295 kPa. Regression analysis showed that the soil cohesion was 22.7 kPa and the internal friction angle was 16.44° under these microscopic parameters. The contact between interfaces also used the parallel bond model. After calculation and debugging, the microscopic parameters of the model are listed in Table 2. Figure 6 shows the deviatoric stress–axial strain curves of soil under varying confining pressures.

Based on the site characteristics observed in the field tests, the ball distribute command was used to generate particles and construct the uplift resistance model. The numbin command was applied to classify the particle gradation. During model construction, a convergence criterion was set, ensuring that the average calculation ratio remained below 1 × 10^−5^ as the equilibrium condition.

**Figure 7:**
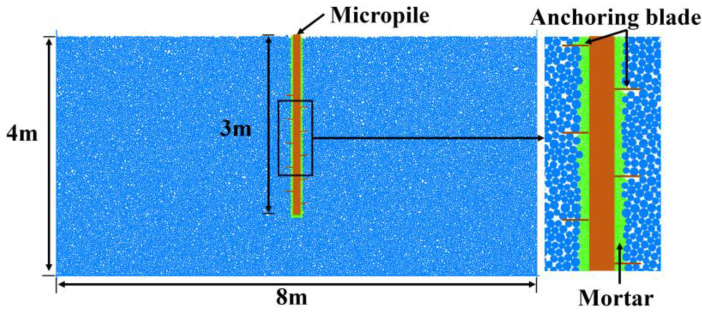
Particle flow model for foundation uplift resistance.

A box-shaped wall structure was established to simulate the anchorage components, with fixed boundary conditions applied to the sides and bottom of the computational domain. The final uplift resistance analysis model, shown in Figure 7, consisted of a total of 34,140 particles. Throughout the simulation, the wall.force.contact and wall.pos commands were used to monitor uplift resistance forces and displacement. The numerical model assumes soil particles as homogeneous spherical particles for mesoscopic parameter calibration. However, when analyzing complex soil strata, repeated calibration of micro-parameters is required, which significantly reduces simulation efficiency.

### 3.4. Numerical Model Validation Results

**Figure 8:**
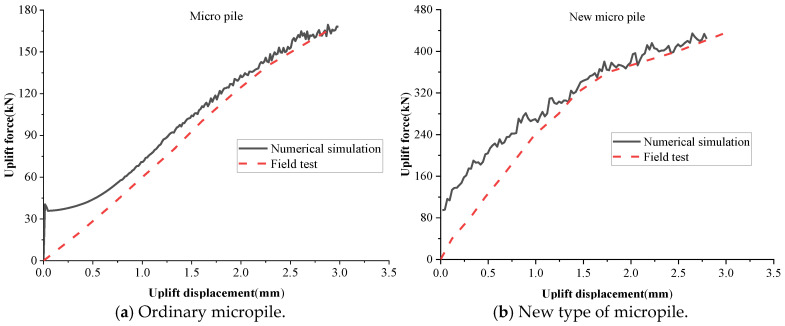
Comparison of numerical model calculation results.

To verify the accuracy and applicability of the micropile uplift resistance model, a comparison was made between the field test data and the numerical simulation results for both conventional micropiles and the novel micropiles, as shown in Figure 8.

The computational results indicate that under high-stress conditions, the numerical simulations closely match the field test results, with a difference rate between the theoretical and the actual values ranging from 8.0% to 12.2%. In particular, for the novel micropile, the load–displacement curves under high-stress conditions exhibit greater consistency between the simulation and experimental results, displaying similar trends and variations. This confirms that the numerical model can accurately simulate the uplift performance of micropiles under high-stress conditions.

However, under low-stress conditions, the numerical model tends to underestimate displacement for the same applied load. This discrepancy is likely due to the computational limitations of the discrete element model, which required particle size scaling for feasibility. As a result, the macroscopic mechanical properties of the model deviate slightly from real-world conditions, with the deviation being more pronounced under low-stress scenarios.

In summary, the numerical model effectively simulates the uplift resistance and displacement behavior of micropiles under high-stress conditions, making it a reliable tool for predicting micropile uplift performance.

### 3.5. Discussion of the Uplift Mechanism of the Novel Micropile

**Figure 9:**
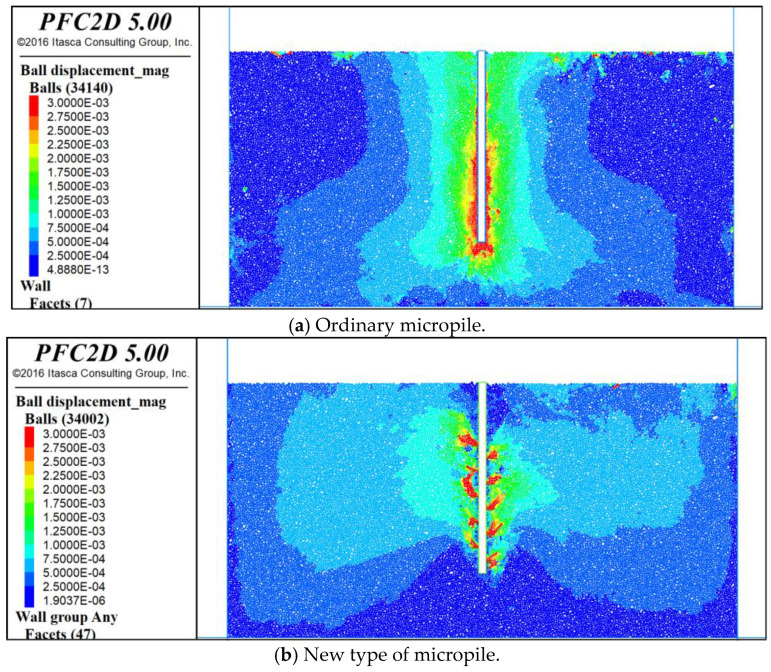
Displacement distribution of soil around pile.

To clarify the uplift mechanism of the new micropile and analyze the displacement changes in the surrounding soil under the load level before reaching the maximum uplift resistance, the field test and numerical simulation results were examined. They showed that the vertical displacement of both micropiles was about 3 mm before approaching the ultimate load. Therefore, the displacement distribution of the surrounding soil under a load of 160 kN for the conventional micropile and 400 kN for the new micropile in the ultimate state was compared, as shown in Figure 9.

**Figure 10:**
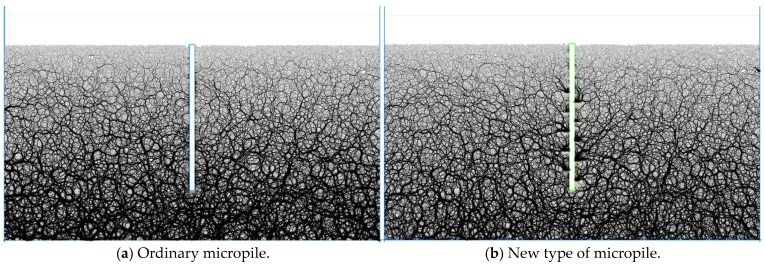
Distribution of force chains in the soil around the pile.

From Figure 9, it can be seen that the new micropile can withstand 2.5 times the uplift resistance of the conventional micropile while effectively controlling the uplift displacement. Within the same displacement field range, the displacement deformation of the new micropile was more concentrated, mainly around the “—”-shaped anchorage components. Moreover, when using the new micropile, the overall displacement of the site soil was significantly smaller than when using the conventional micropile. This is because the anchorage components change the deformation mode of the soil and grout around the pile. In the conventional micropile, the pile provides uplift resistance and generates deformation through friction with the surrounding soil. In the new micropile, deformation is generated through local compressive failure of the soil above the anchorage components, and the anchorage components effectively transfer the uplift load of the pile to the deeper soil. Compared to the widespread frictional deformation between the conventional micropile and the soil, this deformation mode not only enhances the bearing capacity and stability of the pile but also reduces the impact on the surrounding soil. To further clarify the stress distribution around the new micropile, the force chain distribution in the model was analyzed, as shown in Figure 10.

From Figure 10, it can be seen that the force chain distribution in the lower part of the micropile model was relatively uniform, without significant differences. However, around the “—”-shaped anchorage components, vertical and oblique force chains appeared, indicating that the new micropile affects the stress state of the soil around the conventional micropile. Under the uplift force, part of the soil changed from being mainly subjected to vertical interfacial friction to being mainly subjected to anchorage component pressure and micropile interfacial friction, effectively transferring the uplift load of the pile to the deeper soil and thereby significantly enhancing the uplift resistance.

Combined with the field tests, these results show that the introduction of the anchorage components changes the stress state of the soil, increasing the uplift resistance of the new micropile to 2.6 times that of the conventional micropile while effectively controlling the uplift displacement, making the displacement deformation more concentrated and reducing the overall displacement of the site soil. To further optimize the design, the influence of the anchorage component layout range on the uplift resistance of the new micropile was analyzed.

### 3.6. Influence of Anchorage Layout on the Ultimate Uplift Resistance of Micropiles

In the field tests, the anchorage reinforcement length accounted for a relatively high proportion of the total micropile length (pile length = 3 m, anchorage reinforcement length = 2 m). As a result, there are limitations in directly applying these conclusions to longer micropiles.

**Figure 11:**
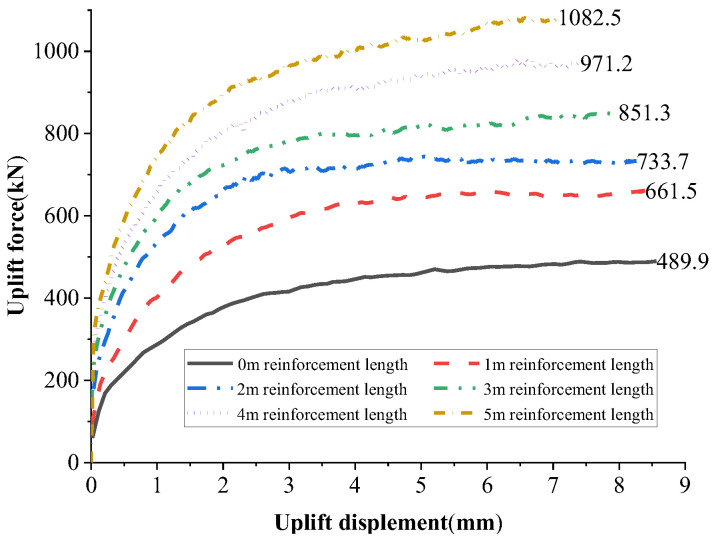
The variation in ultimate tensile capacity of 8 m long micropiles with the reinforcement length of anchors.

To further investigate the impact of anchorage layout on uplift resistance and optimize the design, an 8 m long micropile was used as a case study. Different anchorage reinforcement lengths (ranging from 1 to 5 m) were tested to analyze their effect on the ultimate uplift resistance. The variation in uplift resistance for different anchorage lengths is shown in Figure 11.

As shown in Figure 11, the ultimate uplift resistance of the micropile exhibited a significant upward trend with the increase in the anchorage length of the anchorage components. For an 8 m long micropile without anchorage components, the ultimate uplift resistance was 489.9 kN. When the anchorage length increased from 0 to 1 m, the ultimate uplift resistance rose to 661.5 kN, representing an increase of 35.0%. Subsequently, for each additional meter of anchorage length, the ultimate uplift resistance increased by 10.9% to 16.0%. Ultimately, when the anchorage length reached 5 m, the ultimate uplift resistance of the new micropile significantly improved to 1082.5 kN, marking an increase of 121.0% compared to the conventional micropile.

From the above analysis, it is evident that the new micropile demonstrates a remarkable enhancement in ultimate uplift resistance. Moreover, the anchorage length of the components can be flexibly adjusted according to engineering requirements, achieving a cost-effective reinforcement solution while ensuring stability.

From a cost perspective, the novel micropile design, compared to conventional micropiles, incorporates only a small pneumatic device and an anchoring component in its structural configuration. When the spacing between piles is set to 10 cm, taking an 8 m long micropile as an example, for every additional meter of anchoring length, the construction cost increases by merely 5.7%. However, the ultimate uplift resistance can be enhanced by more than 35.0%.

## 4. Conclusions

This study developed a micropile incorporating a small pneumatic device and an anchoring component. Through field uplift tests and numerical simulations, the following conclusions have been obtained:

1. The novel micropile integrates tightly with the grouting material and surrounding soil as a unified system through a linear anchoring component, significantly enhancing its performance. Field tests indicated that under ultimate conditions, its uplift resistance was improved by 161.7% compared to that of conventional micropiles, while displacement was reduced by 14.7%.
2. A system of vertical and inclined force chains was formed around the anchoring component. By altering the stress distribution within the surrounding soil, the novel micropile converted part of the soil’s load-bearing mechanism under uplift forces from conventional vertical interface friction to a combination of anchoring component compression and micropile interface friction. This transformation significantly enhanced uplift resistance.
3. Numerical simulations showed that without anchoring components, the ultimate uplift resistance of an 8 m long micropile was 489.9 kN. When the anchoring component was introduced with a reinforcement length of 1 m, the ultimate uplift resistance increased to 661.5 kN, representing a 35.0% improvement. When the anchoring reinforcement length was extended to 5 m, the ultimate uplift resistance of the novel micropile reached 1082.5 kN, marking a 121.0% increase.
4. For an 8 m long micropile, every additional meter of anchoring length results in only a 5.7% increase in construction cost, yet the ultimate uplift resistance can be enhanced by more than 35%. This provides an effective treatment approach for addressing engineering challenges related to insufficient anchorage in soil slopes.

## Figures and Tables

**Table 1:** Soil layer physical and mechanical parameter table.

Natural Density/(g·cm^−3^)	Natural Moisture Content /%	Cohesion /kPa	Internal Friction Angle/(°)	Specific Gravity	Liquid Limit /%	Plastic Limit /%
2.0	21.2	22.9	16.3	2.69	29.8	15.6

**Table 2:** Table of numerical simulation micro-parameters.

**Microscopic** **Parameters**	**Particle Density** **/(kg·m^−3^)**	**Particle Size /m**	**Porosity**	**Effective Modulus E/Pa**	**Stiffness Ratio**	**Cohesion /N**	**Coefficient of Friction**
Soil Body	2.0 × 10^3^	0.016~0.024	0.2	1.76 × 10^7^	1.5	1.02 × 10^3^	0.2
Grouting Body	2.5 × 10^3^	0.002~0.003	0.1	2.11 × 10^8^	1.5	1.24 × 10^5^	0.68
Interface	/	/	/	2.9 × 10^5^	1.0	1.94 × 10^3^	0.55

**Figure A1:**
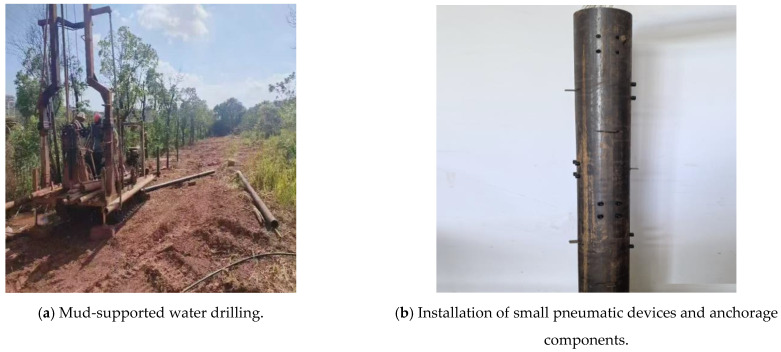

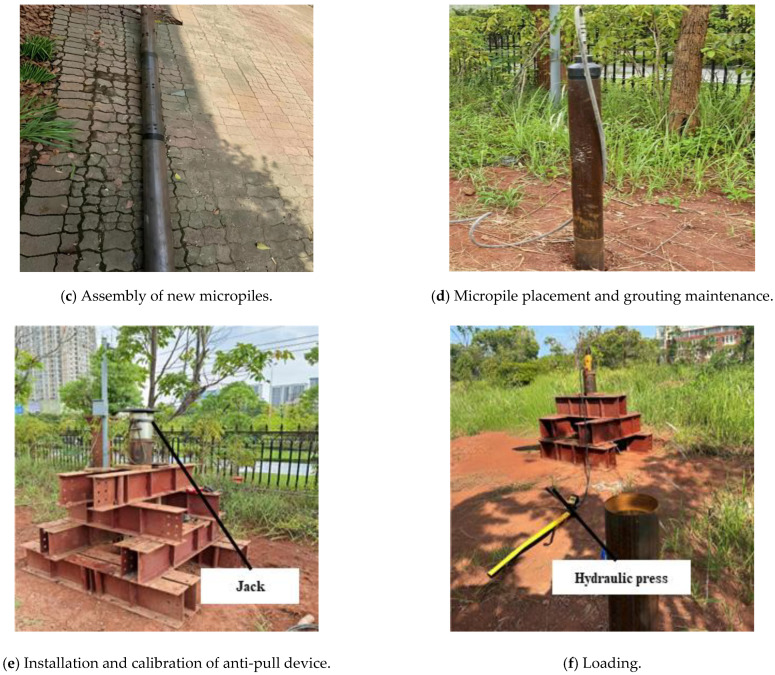
The Process of Field Tension Test for Micropiles.

## Data Availability

The original contributions presented in this study are included in the article. Further inquiries can be directed to the corresponding author.
